# CD55 may be a new target for colorectal cancer treatment

**DOI:** 10.1038/s41598-025-08491-4

**Published:** 2025-07-02

**Authors:** Qiong Luo, Yingjia Mao, Rong Yu, Fuqian Zhao, Hongmei Niu, Daofu Shen, Huan Tian, Lei Wang

**Affiliations:** 1 Third Ward of General Surgery, Department of Affiliated Hengyang Hospital of Hunan Normal University, Hengyang Central Hospital, Hengyang, 421001 Hunan China; 2 Medical Laboratory Center, Chifeng Municipal Hospital/Chifeng Clinical College, Inner Mongolia Medical University, Chifeng, 024000 People’s Republic of China; 3 Changsha Stomatological Hospita, Hunan Unversity of Traditional Chinese Medcine, 389th Youyi Road, Changsha, 410004 China; 4 Hubei Shizhen Laboratory, Hubei University of Chinese Medicine, Wuhan, 430065 China

**Keywords:** Colorectal cancer, CD55, Complement system, ABO blood type, Tumor marker, Cancer, Immunology

## Abstract

In the early stage, our research group injected lentivirus carrying blood type A antigen into tumors as a drug, and the tumor volume of mice was significantly reduced. We speculate that the complement system plays an important role in anti-tumor therapy, but the specific components are unknown. A mouse model containing blood type antibodies was established. After the mouse axilla formed tumors, lentivirus carrying different blood type antigens was used for treatment. On this basis, NK cell activity was inhibited and the complement system of the body was eliminated. Tumor formation was observed. Flow cytometry was used to observe the changes in tumor tissue and peripheral blood immune cells. Immunohistochemistry was used to observe NK cells in tumors. Proteomics was used to screen differential complement components. Transcriptomics was used to observe the gene expression differences of CT26 cells overexpressing CD55, and RT-PCR was used for verification. The changes of CEA, CA199, CA125, CA153, D-D dimer, total white blood cell count, neutrophil ratio, lymphocyte ratio, monocyte ratio, and fibrinogen in the simple colorectal cancer group, simple liver metastasis group, simple lung metastasis group, and multiple organ metastasis group were retrospectively analyzed, and ROC curves were used for evaluation; ELISA method was used to detect the changes in CD55 content in the healthy group, simple colorectal cancer group, simple liver metastasis group, simple lung metastasis group, and multiple organ metastasis group, and ROC curves were used to evaluate its clinical diagnostic ability. CCK8 and EdU methods were used to detect cell proliferation. Scratch assay and transwell assay were used to observe cell migration. Western blotting was used to observe the changes of MMP9, Vimentin and α-SMA. After the lentivirus carrying blood type A antigen was injected into the axillary tumor of mice, the tumor volume was significantly reduced compared with the control group. The proportion of NK cells in tumor tissue increased significantly. Using antibodies to block NK cell surface activation receptors and eliminate the complement system, both treatments reduced the therapeutic effect. Both blood type B and Rh blood type antigens can significantly reduce tumor volume. Eliminating complement in the body increases the incidence of liver metastasis. The proliferation degree of overexpressed CD55 in tumors is significantly increased. CD55 overexpression can significantly increase the expression of CCL2, CCL3, CCL4, CCL7, CXCL12, TLR7, CSFR1, TCAM, TNF, BTK and MMP9. CEA and CA199 are of certain value in judging liver metastasis, and CD55 has better diagnostic efficacy. Complement significantly inhibits cell proliferation. CD55 significantly promotes CT26 proliferation and migration ability. CD55 is a potential tumor promoter and diagnostic marker for colorectal cancer.

**Supplementary Information:**

The online version contains supplementary material available at 10.1038/s41598-025-08491-4.

## Introduction

Colorectal cancer (CRC) is the second most common cancer in women and the third most common cancer in men, and the cancer mortality rate ranks fourth among all tumors^1^. About 80% of colon cancer patients are localized, and the remaining 20% of patients have metastasized to distant sites when they are discovered^2^. For locoregional colorectal cancer, surgical resection is still considered an effective treatment^3^.In the case of liver (or lung) metastasis or locally advanced colorectal cancer, adjuvant chemotherapy is required, but it brings great pain to patients^4^. Radiotherapy or immunotherapy is also an alternative, but the effect is not very ideal^5^. Finding new treatments has always been a long-cherished wish of clinicians.

Our research group previously constructed a mouse model containing blood type A antibodies, and inoculated colorectal cancer and breast cancer in the armpits of the model mice to form subcutaneous tumors^6^. Lentivirus carrying blood type A antigens was used as a drug for intratumoral injection, and the tumor volume of the mice was subsequently significantly reduced. Compared with the control group, the content of C5b-9 complement membrane attack complex in the tumors of mice treated with blood type A antigens increased significantly, and the proportion of NK cells also increased significantly. Therefore, we speculate that complement plays an important role in anti-tumor therapy.

The final result of complement activation is the formation of membrane attack complex, which can create pores in the cell membranes of certain pathogens and body cells, leading to their death^7^. Many diseases caused by abnormal complement regulation can lead to hemolysis and anemia. The most typical example is paroxysmal nocturnal hemoglobin. This is because many autoimmune diseases are characterized by the production of autoantibodies, which bind to host proteins or are deposited in tissues as components of immune complexes^8^. Autoantibodies can activate the complement system, mediating tissue damage and triggering systemic inflammation^9^. In addition, ABO blood type incompatible red blood cell transfusion is often the most common type of hemolytic reaction after mistransfusion. This process is considered to be complement activation, leading to MAC formation and intravascular hemolysis^10^. Complement activation can not only directly promote red blood cell clearance through extravascular and intravascular pathways, but also may cause vascular endothelial damage through the effects of complement split products C3a and C5a on the immune system and vascular permeability, continue to induce extensive thrombin activation, and promote the development of disseminated intravascular coagulation to further aggravate the disease^11^. Due to the limitations of ABO blood type antigens in the treatment of cancer patients, we started with the previous experiments of our research group, took the complement system as an important observation object, and screened out an important target molecule that can be widely used in tumor treatment.

CD55, also known as decay-accelerating factor (DAF), protects cells from complement-mediated attack by degrading C3 and C5 convertases necessary for MAC, after which they can no longer reattach^12^. CD55 was first discovered on the surface of erythrocytes in 1969 and is anchored to the erythrocyte membrane via a glycosylphosphatidylinositol (GPI) linkage^13^. Although primarily localized to the cell surface, soluble CD55 lacking the C-terminal GPI anchor domain has been found in the extracellular matrix, plasma, urine, and body fluids. Cell surface CD55 is a ligand for CD97, which is present on the cell surface of a variety of cell types, including epithelial cells, immune cells, muscle cells, and hematopoietic stem cells^14^. In addition, CD97 is highly expressed in many cancers, including pancreatic cancer, cervical cancer, colon cancer, etc^15^. CD55 can stimulate CD97 to trigger downstream signaling and promote cancer metastasis^16,17^. Epidermal growth factor can induce CD55 mRNA and protein expression in HT-29 colon cancer cells through the p42/44 MAPK pathway^18^. Although there is a view that the destruction of CD55 may have an anti-cancer effect on a variety of cancers, this view is based on the ability to detect a large amount of CD55 expression in tumor tissues, but whether CD55 can be used as a key target in tumor treatment remains to be studied.

In summary, our research was based on the fact that ABO blood type antigens can be used as potential targets for tumor treatment through preliminary experiments. Through proteomics methods, we found that the CD55 content was reduced in the group with good treatment effects. Subsequently, cell experiments proved that CD55 can promote the proliferation and migration of colorectal cancer cell lines, and by collecting clinical samples from colorectal cancer patients, it was proved that it can be used as a potential clinical diagnostic marker. We hope that our research will provide a new idea for the treatment and diagnosis of colorectal cancer.

## Materials and methods

### Establishment of a mouse model containing blood group antibodies

Four-week-old female Babl/c mice were purchased from Sibeifu (Beijing) Biotechnology Co., Ltd. and kept in SPF-grade cages in the Experimental Animal Center of Chifeng Municipal Hospital, with a maximum of 5 mice per cage. 200 µL of 5% red blood cell suspension from Shanghai Blood Biomedical Co., Ltd. was injected intraperitoneally once a week for 3 consecutive weeks. The mice were killed in the fourth week, and whole blood was collected from the mice by eye removal, and peripheral serum was collected. The welfare of experimental animals was carried out in accordance with the Chinese Animal Welfare Guidelines and approved by the Experimental Animal Ethics Committee of Chifeng Municipal Hospital. The study complied with the requirements of the ARRIVE Guidelines 2.0.

### Clinical case collection

Clinical data of CEA, CA199, CA125, CA153, D-D dimer, total WBC count, neutrophil ratio, lymphocyte ratio, monocyte ratio, and Fibrinogen were collected from patients with colorectal cancer in Chifeng Municipal Hospital (*n* = 1008). The patients were divided into: colorectal cancer alone group (*n* = 409), liver metastasis only group (*n* = 249), lung metastasis only group (*n* = 151), and multiple organ metastasis group (*n* = 199). In addition, peripheral sera of 89 patients were collected according to the above groups: health group (*n* = 14),colorectal cancer alone group (*n* = 24), liver metastasis only group (*n* = 23), lung metastasis only group (*n* = 13), and multiple organ metastasis group (*n* = 15). All patients adhered to the Declaration of Helsinki and were approved by the Chifeng Ethics Committee. All patients were familiar with this study and agreed to participate in the study and agreed to the publication of the manuscript.

### CT26 cell culture and lentiviral infection

CT26 is a colon cancer cell line derived from BALB/c mice. All cells were cultured in complete medium consisting of RPMI 1640 (Solerbo, China) containing 10% heat-inactivated FBS (Thermo Fisher, USA), 2 mM glutamine (Solerbo, China), 100 U/ml penicillin and 100 µg/mL streptomycin (Solerbo, China). The cells were incubated at 37 °C in 5% carbon dioxide. The cells were trypsinized and passaged when they reached 100% confluence.The lentiviral vector construct and packaging were provided by Beijing Li Keli Laboratory, and the virus titer was guaranteed to be 1 × 10 ^8^ TU/ml. Tumor cell lines were infected at an MOI of 1:5 for subsequent experiments.

### Establishment of mouse model of liver metastasis

A mouse model of liver metastasis was established. 5 × 10^5^ tumor cells were injected into the spleen of mice. On the 14th day after injection, the mice were killed by cervical dislocation, and the liver specimens were taken for photographing and weighing.

### Lentiviral intratumoral injection therapy

Animals were immunized with ABO blood type antigens for the first 3 weeks. Five days after the tumor cell line (2 × 10^5^/mouse) was inoculated in the mouse armpit, local injection of lentivirus-ABO drugs (1 × 10^7^/mouse, 200 µl) was started, once every 2 days, and the empty virus was used as the control group. In order to prevent the virus drug from entering the blood circulation and reducing the efficacy, the injection method was selected to be carried out around the tumor and multiple points. To ensure the welfare of the mice, when the tumor volume reached 1000 mm^3^ or the maximum tumor diameter reached 15 mm or tumor ulcers appeared, the mice were immediately killed by cervical dislocation. The length and width of the mouse tumor were measured and recorded, and the tumor volume was calculated. The specific formula is: V = length × width^2^ × 0.50.

### NK cell inactivation and complement clearance

In vivo NK cell inactivation was performed using NCR1 Antibody (Affinity Biosciences Cat# DF7599, RRID: AB_2841090, China) diluted 1:10 and injected 100 µl per mouse every three days. In vivo complement clearance was performed using cobra venom factor (Fisher (Hangzhou) Medical Research Co., Ltd., China) injected intraperitoneally at 12.5 µg/mouse /every 2 Days.

### Single cell suspension preparation

A digestion buffer containing 1xDPBS buffer (Lambo Reed, China), collagenase IV (1 mg/ml, Zeye Biotechnology, China), dispase (2.4 mg/ml, Zeye Biotechnology, China), DNase (0.2 mg/ml, Zeye Biotechnology, China) and 3% heat-inactivated fetal bovine serum (Thermo Fisher Scientific, USA) was prepared. The axillary tumors were removed after the mice were killed by cervical dislocation, and the tissue samples were minced into small pieces and placed in 3 ml of digestion solution, and incubated at 37 °C for 30–60 min with slow rotation. The separated cell suspension was passed through a 100 μm cell strainer (BD, USA).

### Peripheral blood PBMC isolation

PBMCs were isolated by centrifugation using Mouse Peripheral Blood Mononuclear Cell Isolation Medium KIT (Tianjin Haoyang, Tianjin). Peripheral blood from each mouse was collected into a tube containing ethylenediaminetetraacetic acid (EDTA), diluted 1:1 with PBS, and the diluted blood was carefully added to the top of the separation medium in two 15 mL centrifuge tubes. After centrifugation at 400× g for 30 min, PBMCs were collected from the middle layer, placed in another centrifuge tube, and washed with PBS. The samples were stored at room temperature for no more than 12 h.

### Flow cytometry

The cell pellet was suspended in 100 µL PBS. The cells were washed once with 500 µL MACS buffer (2 mM EDTA pH8.0, 0.5% BSA in PBS), the supernatant was discarded, and the pellet was suspended in the remaining 50 µL of residual supernatant. To limit non-specific antibody binding, 0.5 µL anti-mouse CD16/32 (BioLegend) was added to the sample on ice for 10 min. Then, the antibody master mix containing 10 µL Brilliant Stain Buffer Plus (BD Biosciences) was added to the sample in 50 µL MACS buffer to a final volume of 100 µL. The samples were placed on ice and stained for 30 min in the dark, then washed with 500 µL MACS buffer. After centrifugation at 500 rcf for 5 min, the samples were suspended in 400 µL MACS and analyzed on a BD FACSymphony A5 cell analyzer. Reagents: CD45-PE (30-F11), CD4-FITC (GK1.5), CD8PE-Cyanine7 (53 -6.7), NK1.1-APC (PK136), all of the above antibodies were purchased from Thermo Fisher Scientific. Analyses were performed using Flowjo software (version: 10.8, https://www.flowjo.com/).

### Immunohistochemistry

Mice were sacrificed, and tumor tissues were obtained and routine immunohistochemistry was performed. Tissue sections were incubated with a 1:100 dilution of the primary antibody of Anti-NK-1R antibody (ab317504, Abcam, UK) at 4 °C overnight. Subsequently, the samples were incubated with a biotinylated secondary antibody (Abcam, UK) at room temperature for 2 h. The color reaction was performed using diaminobenzidine as the enzyme substrate and counterstained with hematoxylin.

### Quantitative real-time PCR

Total RNA was extracted using the RNA extraction kit produced by Beijing Quanshijin Company according to the instructions. Total RNA was reverse transcribed into cDNA using the RR036A reverse transcription kit (TAKARA, Japan). Fluorescence detection was performed using the RR820A qPCR kit (TAKARA, Japan), and data analysis was performed using Bio-Rad software (Version: IQ5, https://www.bio-rad.com/). Relative quantification was performed using the 2^− △△Ct^ method to represent data. GAPDH as the internal reference gene (Table 1).

**Table 1:** Primers used for detection.

Gene name	Upstream primer	Downstream primer
GAPDH	TGTGGGCATCAATGGATTTGG	ACACCATGTATTCCGGGTCAAT
CD55	ACCCCGGTGCATAGAGAAATC	GGATGACGTACTGTTGTCTTGG
CCL2	TTCTTCGATTTGGGTCTCCTTG	GTGCAGCTCTTGTCGGTGAA
CCL3	TGTACCATGACACTCTGCAAC	CAACGATGAATTGGCGTGGAA
CCL4	TTCCTGCTGTTTCTCTTACACCT	CTGTCTGCCTCTTTTGGTCAG
CCL7	CCACATGCTGCTATGTCAAGA	ACACCGACTACTGGTGATCCT
CXCL12	TGCATCAGTGACGGTAAACCA	CACAGTTTGGAGTGTTGAGGAT
TLR2	CTCTTCAGCAAACGCTGTTCT	GGCGTCTCCCTCTATTGTATTG
TLR7	ATGTGGACACGGAAGAGACAA	ACCATCGAAACCCAAAGACTC
CSFR1	AAAGTTTGCCTGGGTCCTCT	TCCTCGGTGATACTCCTGCT
TCAM	TGGTAGAGTTTGGACACTCCC	CTCCCCTGCACAAGAGAAGA
TNF	GCGGCCACAGAAAACACTC	CTCCCAATGGTCAAGGCATC
BTK	TATCAACCGACGGGAGACTGA	TGCCAGTTCGTCTAGTTTCAC
MMP9	GCAGAGGCATACTTGTACCG	TGATGTTATGATGGTCCCACTTG
CXCR3	GGTTAGTGAACGTCAAGTGCT	CCCCATAATCGTAGGGAGAGGT

### CCK8

1 × 10^4^ cells were seeded in a 96-well cell culture plate. The cells were intervened according to the experimental design. Complete medium containing 10 µL CCK8 (Dojindo, Japan) detection reagent was added to each well at different time points (2 h, 4 h, 24 h, 48 h), and the absorbance at 450 nm was measured after 2 h. According to the absorbance at 450 nm, a curve was drawn to estimate the number of cells. The higher the absorbance value, the more surviving tumor cells there are.

### EdU experiment

Take cells in logarithmic growth phase and inoculate them into 96-well plates at 1 × 10^5^ cells per well. After the cells return to normal state, perform the required drug treatment or other stimulation treatment. Add the prepared 10xBrdU solution to the plate wells to obtain a final concentration of 1×. Place the cells in a thermostat. Incubate for 12 h. Remove the culture medium. Add Fixing/Denaturing Solution at 100 µL/well, and place the plate at room temperature for 30 min. Discard the solution. Add the prepared 1xBrdU antibody solution at 100 µL/well, and place the plate at room temperature for 1 h. Discard the solution and wash 3 times with 1×Wash Buffer. Add the prepared 1× HRP-labeled secondary antibody solution at 100 µL/well, and place at room temperature for 30 min. Wash 3 times with 1xWash Buffer. Add 100 µL TMB Substrate and incubate at room temperature for 30 min. Add 100 µL STOP Solution. Read the absorbance at 450 nm.

### Colony formation assay

Treated tumor cells (200 cells) were seeded into six-well plates and incubated for 2 weeks to allow colony formation. Colonies were stained with Giemsa and quantified. Photos were taken at room temperature under a camera (Z30, Nikon).

### Scratch test

Use a ruler to draw horizontal lines at intervals of 0.5 to 1 cm on the back of the 6-well plate, making sure that each well has at least 5 lines passing through it. Add about 5 × 10^5^ cells to each well, making sure that each well is completely covered with cells overnight. The next day, use a pipette tip to draw horizontal lines in each well, keeping the pipette tip vertical and not tilted. Then wash each well 3 times with PBS, remove floating cells, and add serum-free culture medium. Culture in an incubator at 37 °C and 5% CO2. Observe and take pictures under a microscope after 24 h.

### Transnswell experiment

Digest the cells with trypsin and wash twice with PBS. Resuspend the cells in serum-free medium and adjust the cell density to 5 × 10^5^ cells/mL. Add 200 µL of cell suspension to the upper chamber of the Transwell chamber, and add 700 µL of medium containing 10% FBS to the lower chamber of the 24-well culture plate. Place the culture plate in a 37℃ CO2 incubator for 24 h, remove the cells, and wash twice with PBS. Fix the cells in another well plate with methanol solution for about 30 min, and then stain with crystal violet solution for 15 min. Carefully wipe off the cell residue from the upper layer of the microporous membrane with a cotton swab, and finally take pictures under an inverted microscope.

### Western blotting

Proteins were routinely extracted and 20 µg was added to each well. The proteins were separated by 10% SDS-PAGE and transferred to polyvinylidene difluoride membranes. The membranes were blocked with 5% skim milk and then incubated with primary antibodies overnight at 4 °C. Subsequently, the membranes were incubated with horseradish peroxidase-conjugated secondary antibodies for 2 h. The blots were developed using the ECL system. MMP9 (Cat# AF5228, RRID: AB_2837714), alpha-SMA (Cat# AF1032, RRID: AB_2835329), Vimentin (Cat# BF8006, RRID: AB_2847777), GAPDH (Cat# T0004, RRID: AB_2833041) were purchased from Affinity Biosciences. Using Image J software (Version: 1.8.0, https://imagej.net/software/imagej/), the exposure images were converted into 8-bit grayscale images and normalized with GAPDH.

### ELISA

According to the instructions of the mouse CD55 kit (Keborui, China), the peripheral blood CD55 content was calculated by colorimetry using an ELISA reader (450 nm). According to different groups, the ROC curve method was used to evaluate its ability to be used as a clinical diagnostic marker.

### Statistical analysis

Statistical analysis was performed using GraphPad Prism software(Version: 11.0, https://www.graphpad.com/). The results are presented as the mean ± standard error of the mean (SEM) of at least three independent samples. The Student’s t-test was used to analyze the differences between the two groups. One-way ANOVA was used when there were more than two groups. The differences were considered significant when the *P* value was < 0.05.

## Results

### The complement system plays an important role in anti-tumor

**Fig. 1:**
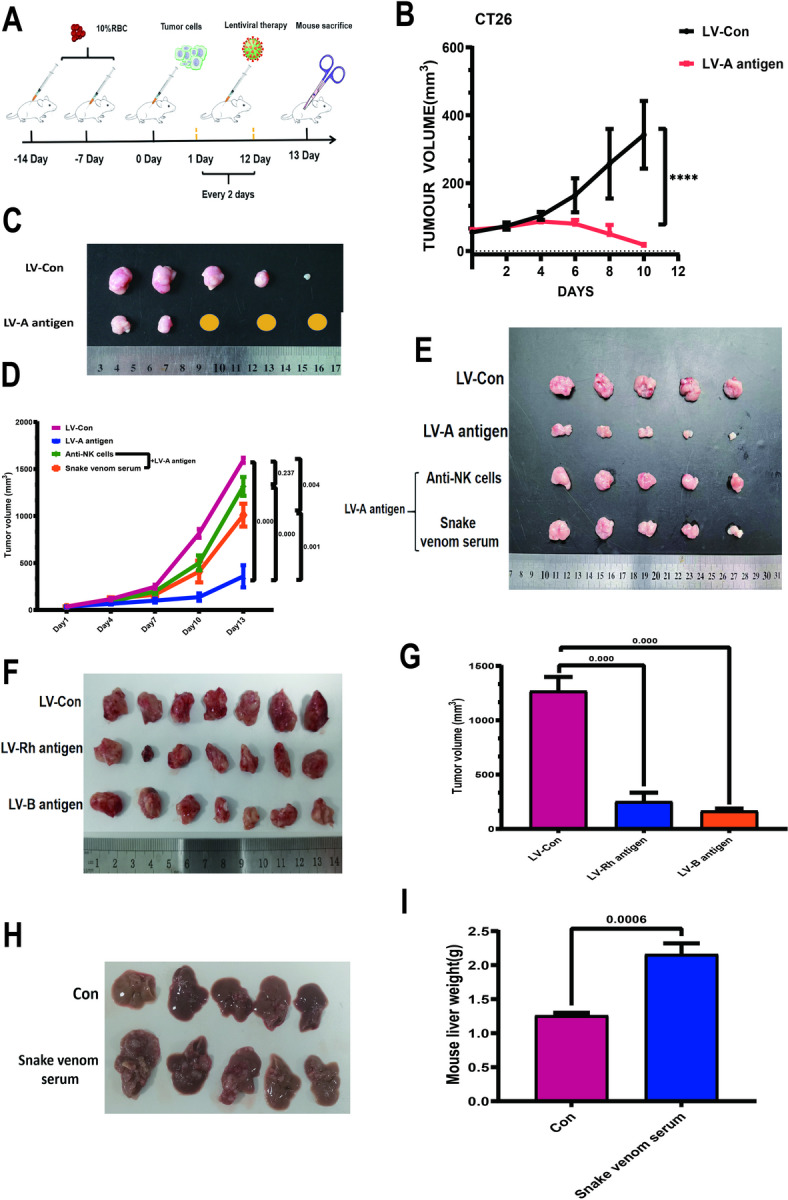
The complement system plays an important role in anti-tumor. (A) Modeling flow chart. 14 days before axillary tumor injection, 10% red blood cell suspension of different blood types was intraperitoneally injected every week. On the third day after axillary tumor injection, lentivirus carrying blood type antigen was used for treatment, and treatment was performed once every 2 days. Mice were killed after 13 days. The flow chart was drawn using WPS Office (PC version 2021, China). (B) Tumor growth curve. Tumor volume was calculated as V = length x width^2^ × 0.50. Student’s t test was used for testing, and the results are expressed as mean ± SEM, ****P < 0.0001. (C) Mouse tumor images (*n* = 5 per group). (D, E) Mice producing blood type A antibodies were constructed and then treated with: (1) control virus treatment; (2) lentivirus carrying blood type A antigen treatment; (3) lentivirus carrying blood type A antigen treatment and blocking NK cell activity; (4) lentivirus carrying blood type A antigen treatment and clearing complement. NK cell blocking was performed using NCR1 Antibody. Snake venom serum was used for complement removal, and the dosage was 12.5 µg/mouse /every 2 Days. The tumor diameter was measured and the volume was calculated on days 1, 4, 7, 10, and 13. The tumor was photographed after sacrifice. *n* = 5 in each group. *P* < 0.05 was considered significant. (F, G) The effect of B and Rh blood type antigens on tumor treatment was observed according to the principle of A blood type antigen (*n* = 7 in each group). The tumor was photographed and the volume was calculated after the mice were sacrificed. *P* < 0.05 was considered significant. (H, I) CT26 tumor cells 5 × 105/mouse were injected into the spleen of mice to simulate the liver metastasis model of colorectal cancer. Snake venom serum was used to remove complement in mice from the second day. The dosage was 12.5 µg/mouse /every 2 Days. The mice were sacrificed on the 14th day, and the liver tissue was photographed and weighed (*n* = 5 in each group). *P* < 0.05 was considered significant.

According to the previous results of the research group, we reconstructed a mouse model that can produce blood type antibodies and used the corresponding blood type antigen for treatment. The day of tumor injection was defined as day 0 (Fig. 1A). When mice were immunized with blood type A antigen, the tumor volume was significantly reduced compared with the control group after the lentivirus carrying blood type A antigen was injected into the mouse axillary tumor. The tumor disappeared in 3 mice (Fig. 1B, C). We detected immune cells related to anti-tumor. After treatment with blood type A antigen, the proportion of NK cells in tumor tissue increased significantly (Fig. Supp1A), but there was no significant change in peripheral blood (Fig Supp1B). Immunohistochemistry was used to prove again that NK cells in tumor tissue increased significantly after treatment with blood type A antigen (Fig Supp1C). NK cells are considered to be the most important cell population for ADCC action of the complement system. In order to speculate on the role of complement, we used antibodies to block the activation receptors on the surface of NK cells and clear the complement system, and found that both treatments reduced the therapeutic effect of blood type A antigen, accelerating tumor growth (Fig. 1D, E). We also observed that B blood type and Rh blood type antigens can also be used as a strategy for tumor treatment, and both can significantly reduce tumor volume (Fig. 1F, G). Colorectal cancer patients are prone to liver metastasis, and we found that clearing complement in the body can significantly increase the occurrence of colorectal cancer liver metastasis (Fig. 1H, I).

### CD55 promotes tumor growth in mice

**Fig. 2:**
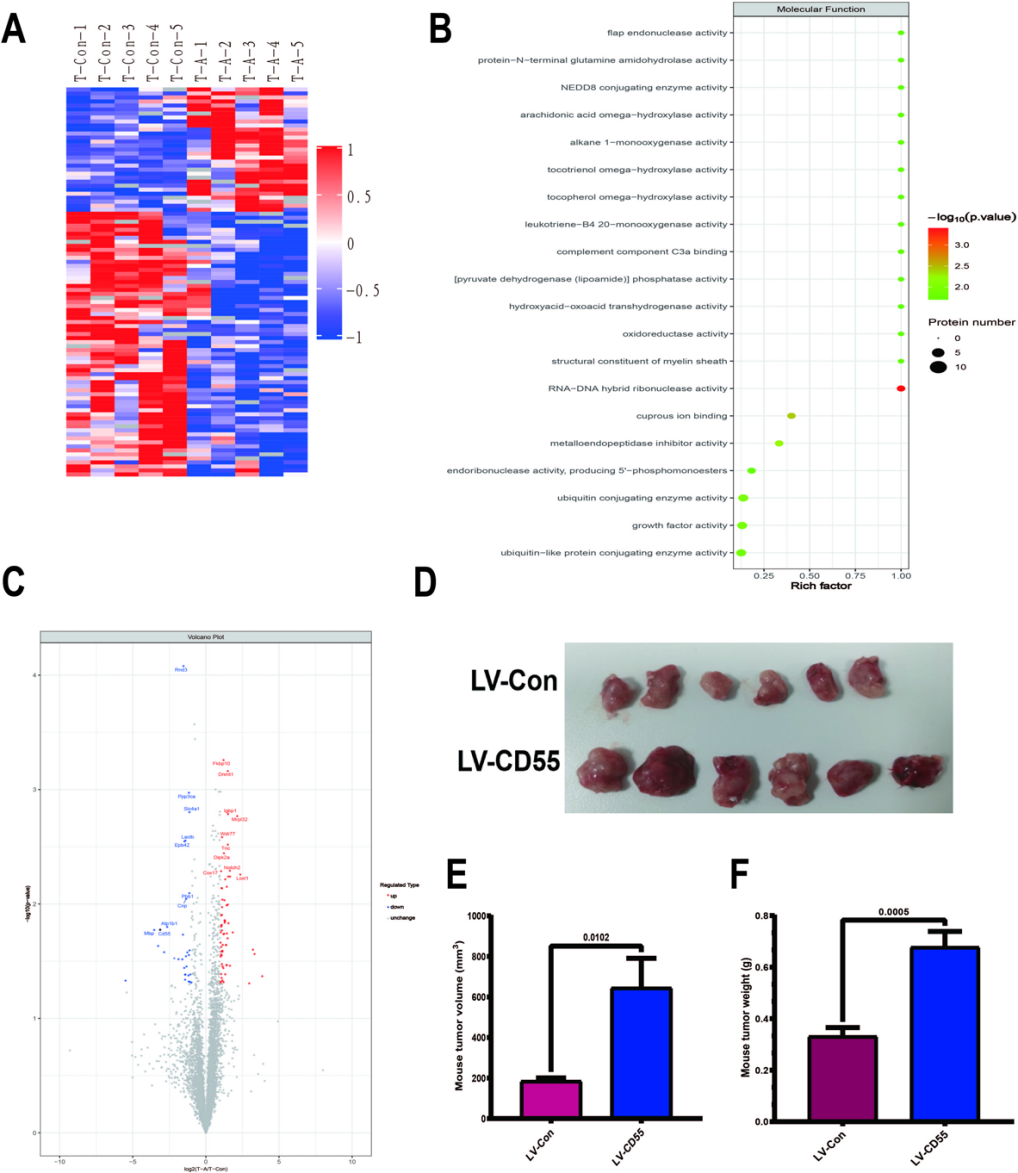
CD55 promotes tumor growth in mice. 5 × 10^5^ CT26 tumor cells were injected into the axilla of mice expressing blood type A antibodies, and 3 days later, they were treated with lentivirus carrying blood type A antigen. Mice were killed on the 13th day, and tumor tissues were removed and tested using proteomics (*n* = 5 per group). Lentivirus without any gene was used as the control group. (A) Heat map between the two groups shows the expression changes of different proteins. (B) GO analysis. (C) Volcano map. (D–F) Complement component CD55 was initially screened. CD55-expressing lentivirus was constructed. Intratumoral injection of mouse axillary tumors was performed (same method as before). Mice were killed on the 13th day, tumor tissues were photographed, and the volume and tumor weight were measured (*n* = 6 per group). *P* < 0.05 was considered significant.

In order to find the complement involved in the anti-tumor effect of blood type A antigen, the tumor tissues of the control group and after blood type A antigen treatment were detected using proteomics technology, and the protein composition of the two tumor tissues changed significantly (Fig. 2A). GO analysis revealed that complement C3 was involved in the anti-tumor effect (Fig. 2B). By combing all proteins, it was found that CD55 was significantly reduced after blood type A antigen treatment, and it was the only protein related to complement (Fig. 2C). Subsequently, we constructed a mouse axillary tumor model and used a lentivirus carrying CD55 for intratumoral injection. Compared with the control group, the proliferation rate of tumors overexpressing CD55 increased significantly (Fig. 2D–F).

### CD55 modifies tumor cell heterogeneity

**Fig. 3:**
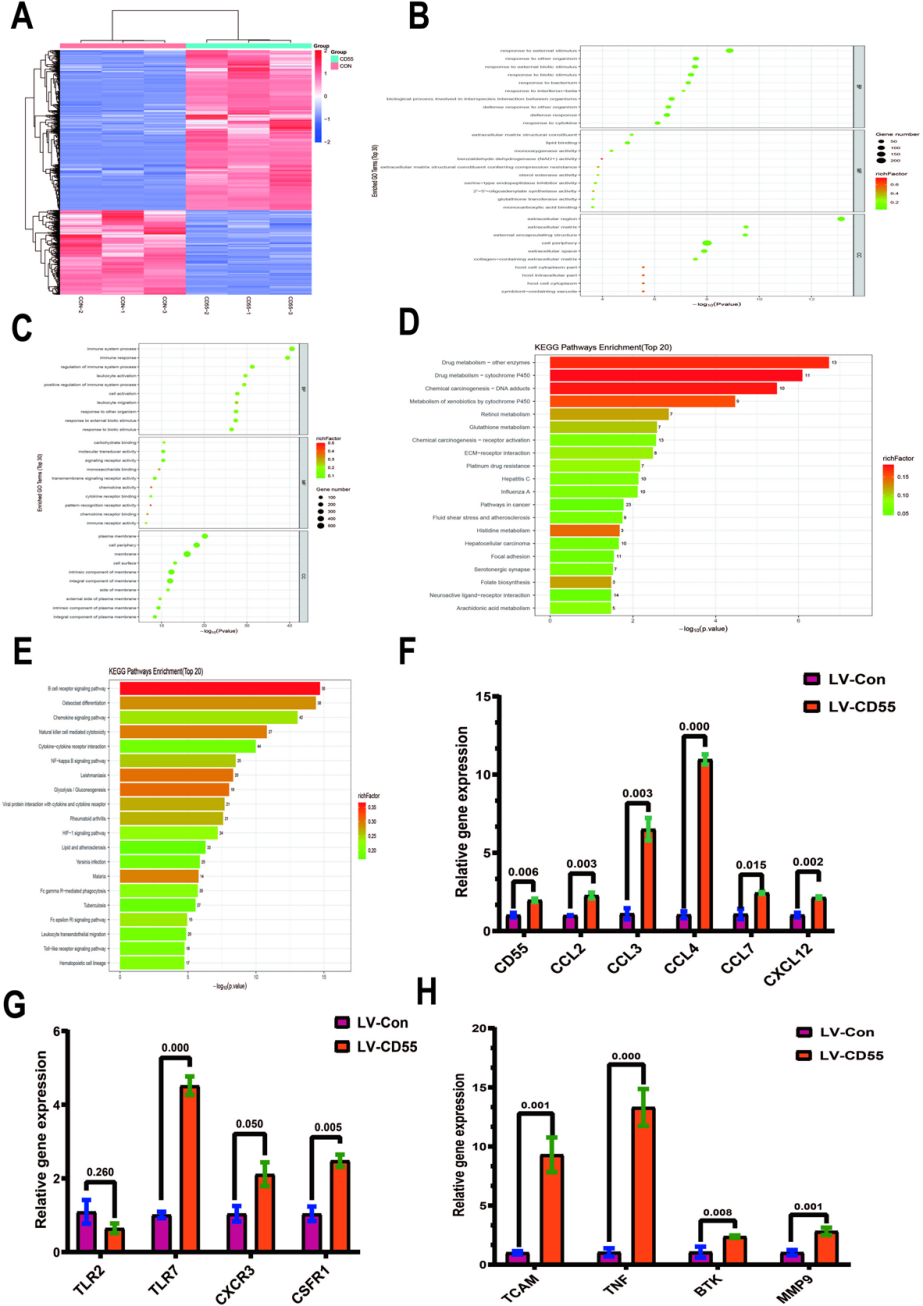
CD55 modifies tumor cell heterogeneity. CT26 cells were routinely cultured and infected with a CD55-expressing lentivirus at an MOI of 1:5 for transcriptomic analysis. Empty virus was used as the control group. (A) Heat map. (B) GO analysis of downregulated genes. (C) GO analysis of upregulated genes. (D) KEGG analysis of downregulated genes. (E) KEGG analysis of upregulated genes. (F–H) Quantitative Real-time PCR was used to verify the transcriptomic results. GAPDH was used as an internal reference, and each group was repeated 3 times. Student’s t test was used for testing, and the results are expressed as mean ± SEM. *P* < 0.05 was considered significant.

We are very concerned about the effect of CD55 on tumor cells. Therefore, we infected CT26 cells with CD55 lentivirus and detected the transcriptome. We found that overexpression of CD55 can significantly change the gene expression of CT26 cells (Fig. 3A). Using GO analysis, we analyzed the downregulated genes (Fig. 3B) and upregulated genes (Fig. 3C), respectively, and found that the immune defense-related processes increased significantly. Using KEGG analysis, we analyzed the downregulated genes (Fig. 3D) and upregulated genes (Fig. 3E), respectively, and found that the genes related to B cells, NK cells and chemokines increased significantly. According to the sequencing results, we verified according to chemokines (Fig. 3F), receptors (Fig. 3G) and immune-related factors (Fig. 3H). Overexpression of CD55 can significantly increase the expression of CCL2, CCL3, CCL4, CCL7, CXCL12, TLR7, CSFR1, TCAM, TNF, BTK and MMP9.

### CD55 has diagnostic value for colorectal cancer metastasis

**Fig. 4:**
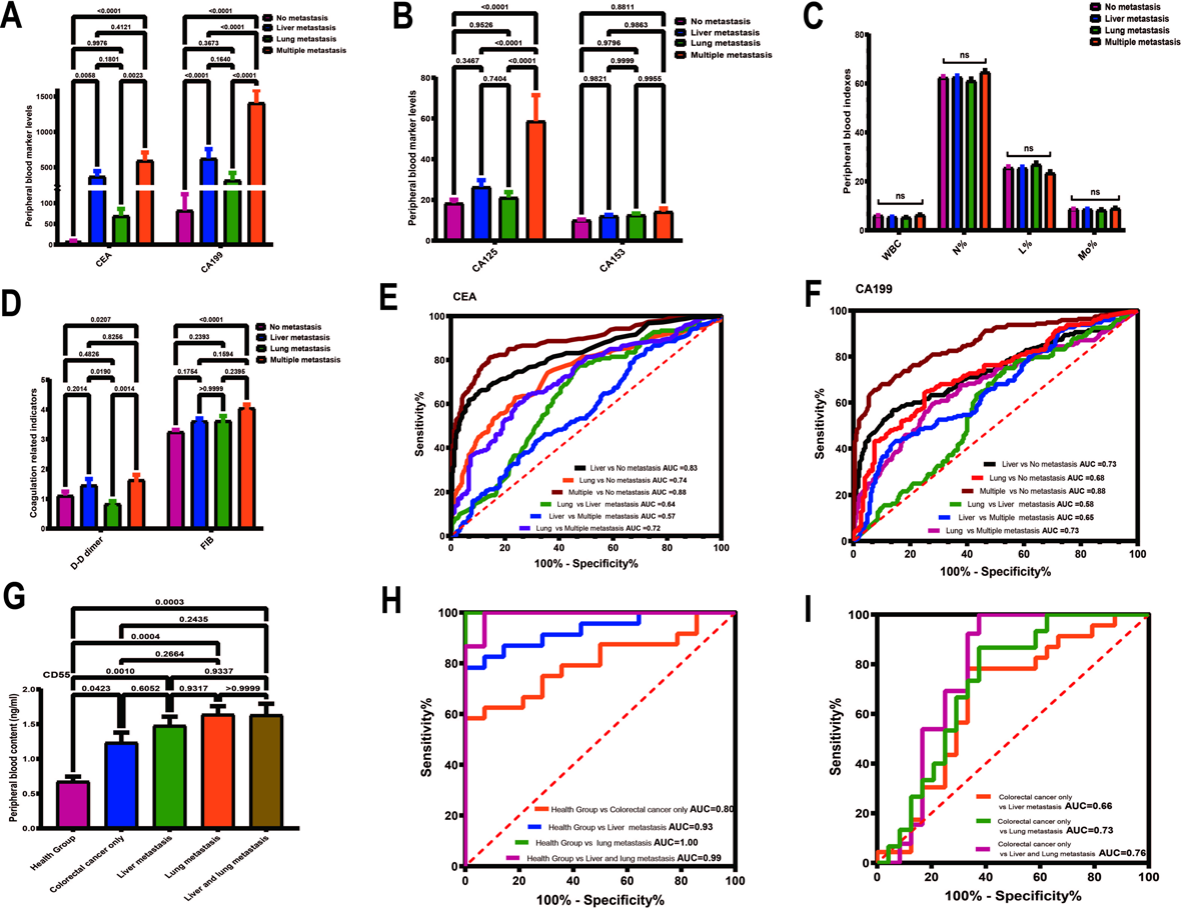
CD55 has diagnostic value for colorectal cancer metastasis. The relationship between clinical test results and tumor metastasis in patients with colorectal cancer was retrospectively analyzed. Data of 1008 patients in Chifeng Municipal Hospital were collected. According to the pathological type, the patients were divided into: simple colorectal cancer group (409 cases), simple liver metastasis group (249 cases), simple lung metastasis group (151 cases), and multiple organ metastasis group (199 cases). (A) Expression of CEA and CA199 in different groups. (B) Expression of CA125 and CA153 in different groups. (C) Changes in white blood cell count, neutrophil ratio, lymphocyte ratio and monocyte ratio. (E) Determination of D-dimer and fibrinogen content. (E, F) The ROC curve method was used to observe the diagnostic efficacy of CEA and CA199 as diagnostic markers in different groups. (G) Peripheral blood samples of 89 subjects were collected and divided into health group (*n* = 14), colorectal cancer alone group (*n* = 24), liver metastasis only group (*n* = 23), lung metastasis only group (*n* = 13), and multiple organ metastasis group (*n* = 15). The Elias method was used to detect the content of CD55 in peripheral blood. (H, I) The ROC curve method was used to observe the diagnostic efficacy of CD55 as a diagnostic marker among different groups. One-way ANOVA was used when there were more than two groups. The differences were considered significant when the P value was < 0.05. International units of tumor markers: CEA (ng/ml); CA15-3; (U/mL); CA125 (U/mL); CA199 (kU/L). Abbreviation: WBC (White Blood Cell, unit: 109/L); FIB (fibrinogen, unit: g/L); N% (neutrophil ratio); L% (lymphocyte ratio); M% (monocyte ratio).

Valuable tumor markers are very helpful for tumor prognosis. In order to determine the value of CD55 as a tumor marker. We first retrospectively analyzed the commonly used clinical test indicators in patients with colorectal cancer. These patients were divided into: colorectal cancer alone group, liver metastasis only group, lung metastasis only group, and multiple organ metastasis group. Compared with the colorectal cancer only group, CEA and CA199 in patients with liver metastasis increased significantly, while CA125 and CA153 did not change significantly (Fig. 4A, B). Other indicators such as D-D dimer, total WBC count, neutrophil ratio, lymphocyte ratio, monocyte ratio and Fib had no diagnostic value (Fig. 4C, D). The ROC curve method was used to prove again that CEA and CA199 have certain value in judging colorectal liver metastasis (Fig. 4E, F). We then collected serum from some clinical patients and divided them into health group, colorectal cancer alone group, liver metastasis only group, lung metastasis only group, and multiple organ metastasis group. As long as colorectal cancer occurs, the content of CD55 increases significantly regardless of whether there is metastasis, and it has a very effective diagnostic efficacy (Fig. 4G, H). Compared with colorectal cancer alone, CD55 also has a certain diagnostic ability for detecting organ metastasis (Fig. 4I).

### CD55 increases the proliferation and migration ability of colorectal cancer

**Fig. 5:**
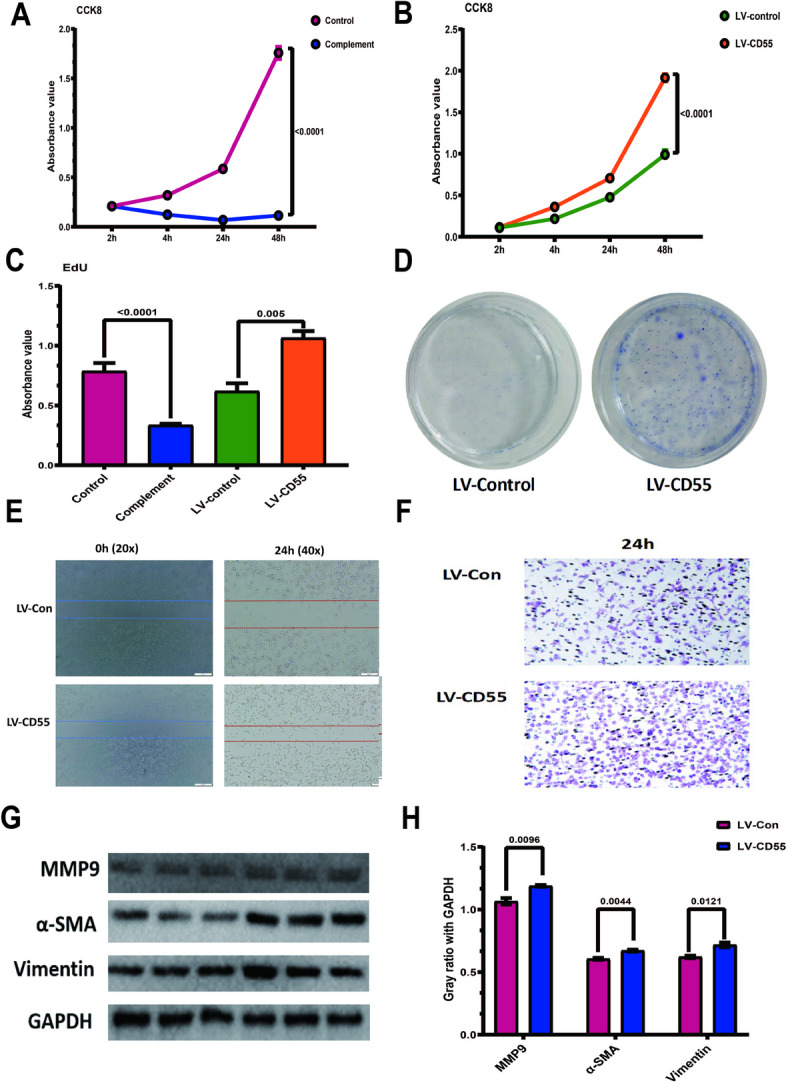
CD55 increases the proliferation and migration ability of colorectal cancer. Conventional culture of CT26 cells. Add 1 × 104/well cells to a 96-well plate. Add guinea pig serum to a concentration of 5%. Use a medium containing 10% FBS as a control. Use the CCK8 method to measure the absorbance at 450 nm for 2 h, 4 h, 24 h, and 48 h. (B) Use CD55-carrying lentivirus to infect CT26 cells. Add 1 × 104/well cells to a 96-well plate. Use the CCK8 method to measure the absorbance at 450 nm for 2 h, 4 h, 24 h, and 48 h. Use an empty virus as a control. (C) Repeat the above experiment using the Edu method. (D) Use CD55-carrying lentivirus to infect CT26 cells. Evenly place 200 cells in a small dish. Stain with Giemsa 2 weeks later and observe the results. (E) Use CD55-carrying lentivirus to infect CT26 cells. Add the cells to a small dish to ensure 5 × 105/dish. On the second day, a horizontal line was drawn in each well with a pipette tip, and the cells were observed and photographed under a microscope 24 h later. (F) 1 × 104/well was added to the upper chamber of the Transwell chamber, and crystal violet was used to stain and photographed 24 h later. (G, H) CT26 cells were infected with CD55 lentivirus, and the expression of EMT-related proteins was detected by Western Blot. GAPDH was used as an internal reference to analyze the relative gray value. The above experiments involved the sampling of quantitative data with mean ± SEM, and Student’s t-test was used for statistical analysis. At least three replicates were required for each group. *P* < 0.05 was considered statistically significant.

Complement usually has a killing effect on cells. Guinea pig serum was used as a complement source. Complement significantly inhibited cell proliferation compared with the control group without complement (Fig. 5A). CD55 was then overexpressed in the CT26 cell line using lentiviral infection. Compared with the control group, CD55 significantly increased the proliferation of CT26 (Fig. 5B–D). Using scratch assays and Transnswell, it was demonstrated that CD55 significantly increased the migration ability of CT26 (Fig. 5E, F). Subsequently, the three proteins MMP9, Vimentin and α-SMA related to EMT were detected, and they all increased significantly after CD55 overexpression (Fig. 5G, H, Supp2).

## Discussion

The incidence of colorectal cancer is on the rise, bringing heavy medical and health burdens to the world and China^19^. Our research group has found a new treatment strategy for colorectal cancer in previous studies, but the mechanism remains to be explored. This study proves that the complement system plays an important role in colorectal cancer and found that CD55 can promote colorectal proliferation and metastasis, which may also become a potential tumor marker for the diagnosis of colorectal cancer.

The complement system was discovered in 1890 and was considered to be a heat-labile component in serum with antibacterial effects^20^. With the continuous development of knowledge, it has been determined that the complement system is an important component of the innate immune system. The complement system is mediated by more than 50 blood circulation or cell surface binding proteins and is involved in a variety of physiological processes such as regulating adaptive immunity, enhancing humoral immunity, angiogenesis and tissue regeneration^21^. Varga et al. found that complement levels are related to the opsonization of NK cells^22^. Therefore, complement has always been considered to have anti-tumor effects. Consistent with our study, the proportion of NK cells in tumor tissue increased significantly after effective tumor treatment. Nishioka et al. found that the level of complement protein in the plasma of lung cancer patients was significantly higher than that of the control group, and the increased complement level was related to the size of lung tumors^23^. In patients with chronic lymphocytic leukemia, it was found that low activity of the classical complement pathway predicted short survival in patients with chronic lymphocytic leukemia^24^. However, the complement system has a complex role in the tumor microenvironment. Recent studies have shown that the complement system may be related to tumor promotion. Markiewski et al. observed that C5a was released into the tumor microenvironment due to complement activation, thereby recruiting and activating myeloid-derived suppressive immune cells to reduce T cell anti-tumor responses^25^. In many tumor types (lung cancer, breast cancer, pancreatic cancer, kidney cancer), complement proteins C1q, C1s, C3, C4, anaphylatoxins C3a and C5a and their receptors and regulatory proteins (such as factor B, factor H, factor I, CD55 and CD59) are most often overexpressed^26–29^. Therefore, it has been proposed that local complement activation within the tumor may play a role in tumor progression. Our study has shown that a single complement component CD55 can significantly increase the proliferation and metastatic capacity of tumors. Interestingly, after the use of snake venom serum to remove the body’s complement, the ability of colorectal cancer to metastasize to the liver increased, while the effect of tumor treatment could be reduced. Therefore, the role of the complex complement system in tumor treatment needs to be further studied.

Since CD55 was discovered on the surface of erythrocytes, many studies have focused on the role of CD55 in immune surveillance, and all believe that it plays a key role in complement regulation^30^. It is now increasingly recognized that CD55 plays an important role in cancer. Growth factors and cytokines produced by tumors can induce CD55 gene and protein expression^31^. Epidermal growth factor can induce CD55 expression in colon cancer cells through the p42/44 MAPK pathway^17^. Similarly, vascular endothelial growth factor can induce CD55 expression in HUVEC cells^32^. Cytokines such as TNF-α, IL-6, and IL-1β can increase CD55 expression in liver cancer cells^33^. Reducing the expression of CD55 can have an anti-cancer effect on a variety of cancers (including ovarian cancer, cervical cancer, and breast cancer)^34–36^. Similarly, Bharti et al.^37^ found that CD55 promotes chemoresistance and the increase of cancer stem cells by activating ROR2/JNK signaling and upregulating SOX2, Nanog, and OCT4 expression in endometrioid ovarian cancer. Dho et al.^38^ even developed a radionuclide-labeled monoclonal anti-CD55 antibody with therapeutic diagnostic potential for pleural metastatic lung cancer. Although we did not observe the efficacy of reducing CD55 expression in mouse tumors, our study found that CD55 can be a potential tumor marker for the diagnosis of colorectal cancer.

Cell surface glycoproteins play a key role in cell function, including intercellular communication, cell-extracellular matrix interaction, and cell response to environmental factors, and can even regulate antigen presentation and initiate immune signaling pathways^39^. N-glycosylation occurring on asparagine is the most common and important protein modification in most organisms^40^. Abnormal glycosylation is considered one of the hallmarks of cancer. Abnormal glycosylation usually includes changes in glycoprotein expression levels caused by abnormal glycosylation modification and changes in glycoprotein sugar structure determined by glycogen changes^41^–43). ABO blood group is one of the most important blood group systems in humans. ABO (H) antigens were found to be terminal non-reducing epitopes of large complex erythrocyte N-glycans, and also constitute a unique feature of the N-linked structure of lower molecular weight outer arm glycoproteins^44^. The ABO gene encodes two specific glycosyltransferase alleles (i.e., A and B), which can catalyze the covalent attachment of N-acetyl-D-galactose or D-galactose to the common precursor side chain (i.e., H antigen), ultimately forming A and B antigens, respectively^45^. Although the specific mechanism is not well understood, our research group previously used lentivirus expressing blood type A antigen to treat tumors and significantly reduced tumor volume. In this experiment, we used blood type B antigen and Rh blood type antigen to achieve the same effect.

This study still has some limitations. When studying the clinical diagnostic application of CD55, there is a clear lack of large cohort samples, which can serve as the focus of subsequent research by our research group. In short, our study has proved that blood type antigens can be potential targets for tumor treatment. The complement system plays a key role in anti-tumor. CD55 is a potential tumor promoter and diagnostic marker for colorectal cancer.

## Electronic supplementary material

Below is the link to the electronic supplementary material.

Supplementary Material 1

Supplementary Material 2

Supplementary Material 3

## Data Availability

The datasets used and/or analysed during the current study available from the corresponding author on reasonable request.
