# Supplementary figures and images for: CD55 may be a new target for colorectal cancer treatment
Source: Sci Rep. 2025 Jul 2;15:23086. doi: 10.1038/s41598-025-08491-4 (PMC12216819; doi:10.1038/s41598-025-08491-4)

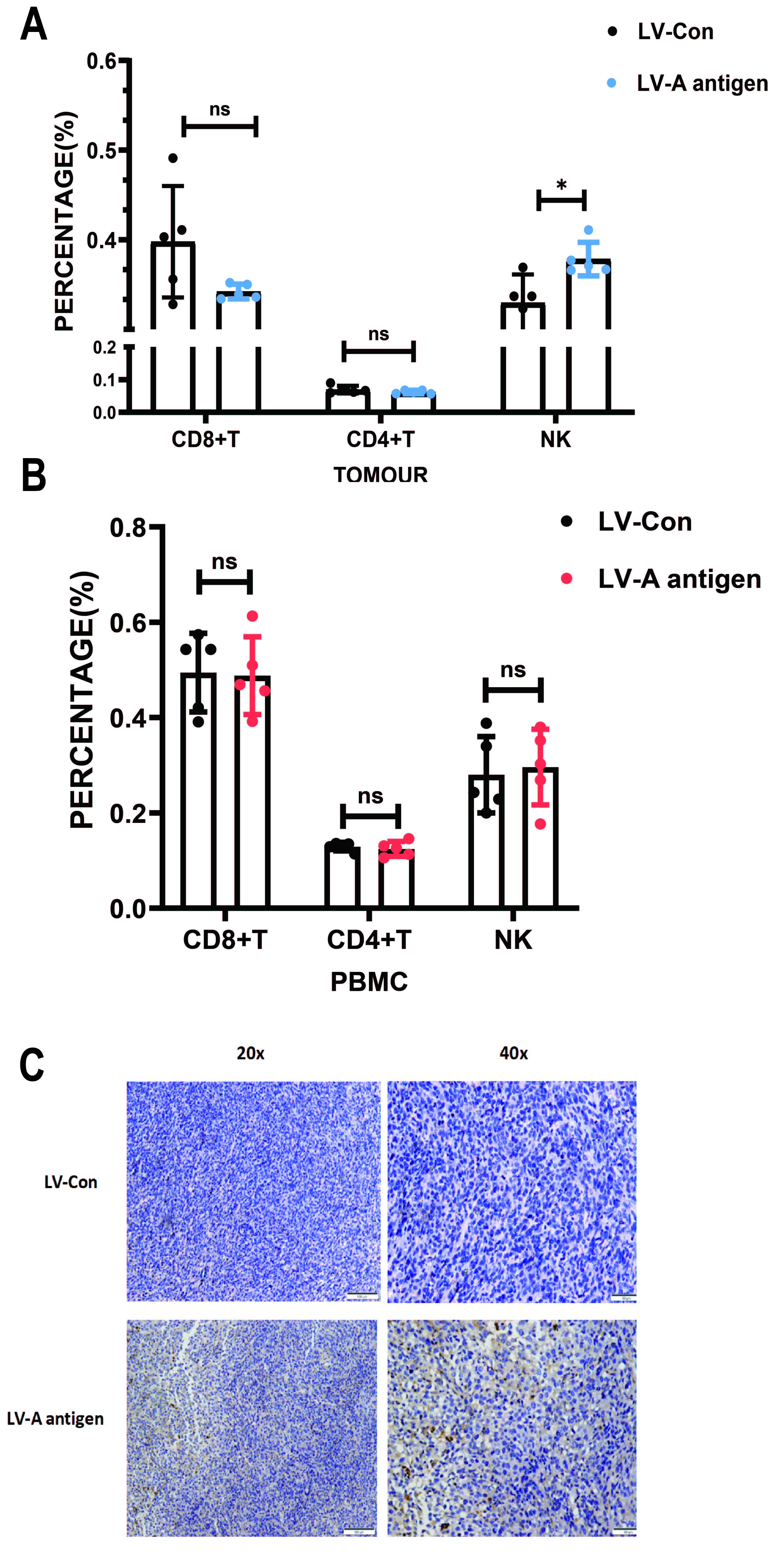

Supplement: Supplementary file 1 — Supplementary Material 1 [file 41598_2025_8491_MOESM1_ESM.jpg]

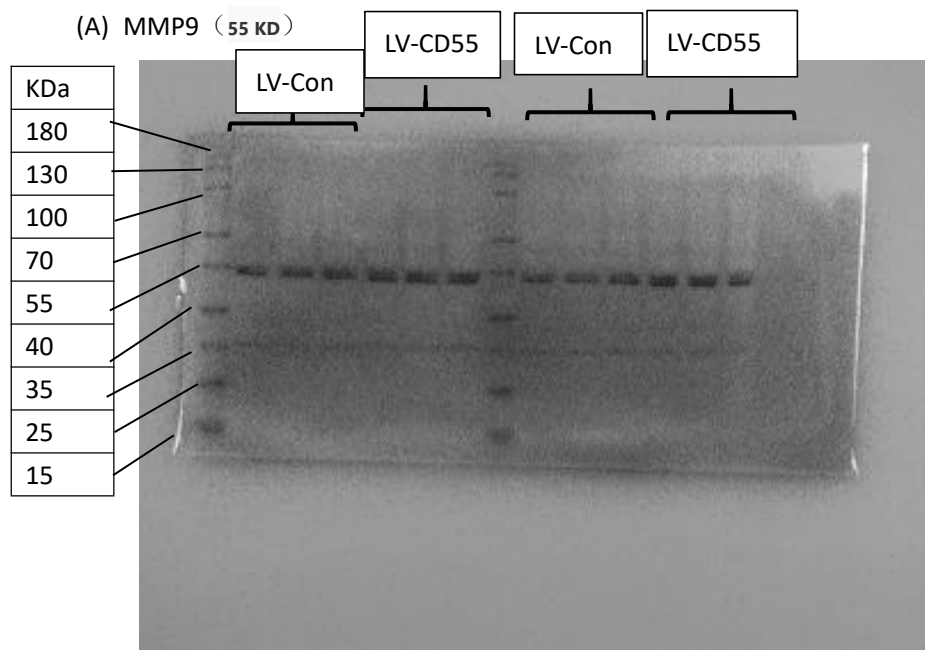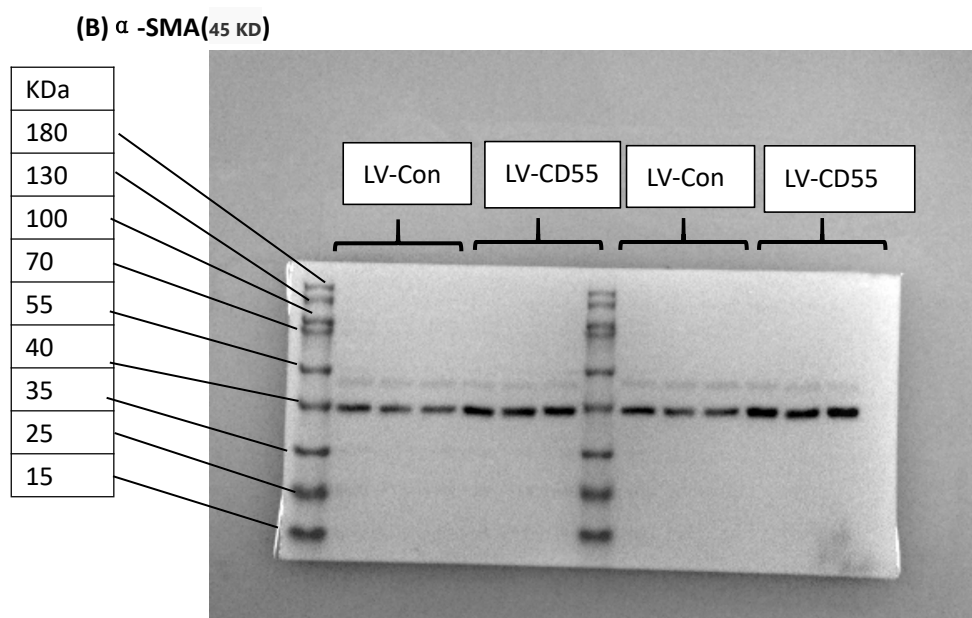

(C)Vimentin(53kDa)

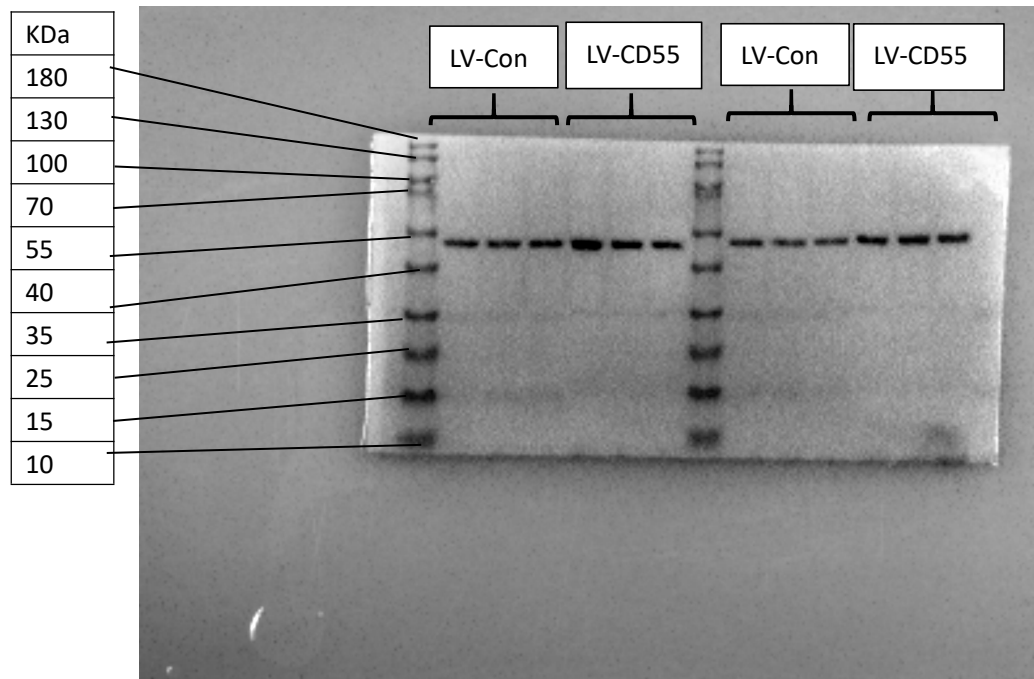

(D)GAPDH(37 KD)

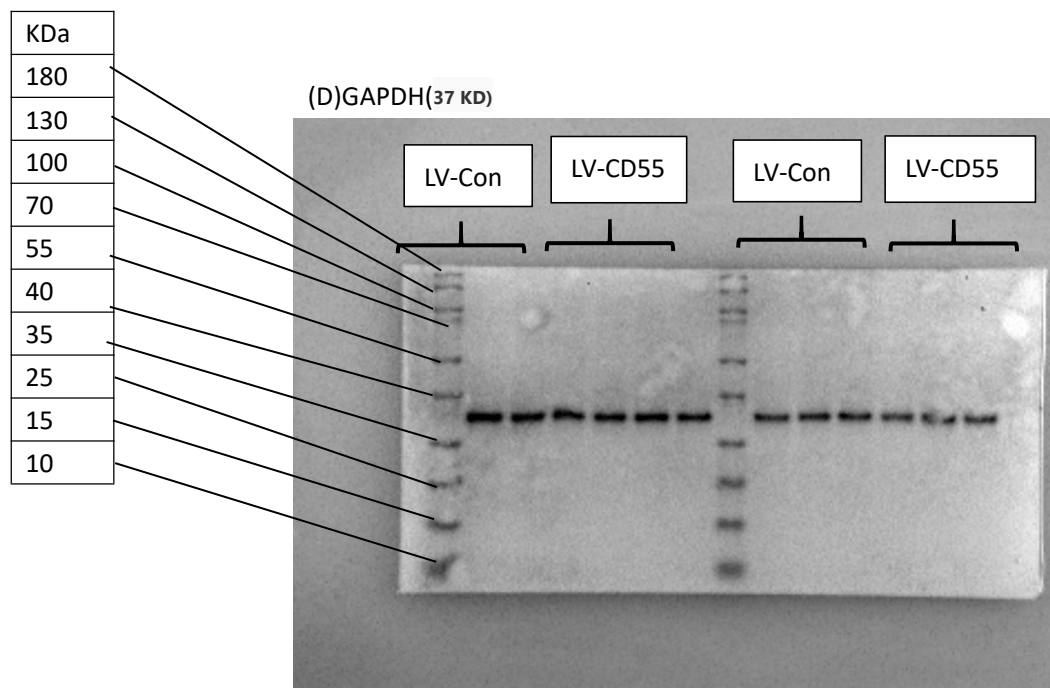

Supplement: Supplementary file 2 — Supplementary Material 2 [file 41598_2025_8491_MOESM2_ESM.pdf]
