# Supplementary material for: CD55 may be a new target for colorectal cancer treatment
Source: Sci Rep. 2025 Jul 2;15:23086. doi: 10.1038/s41598-025-08491-4 (PMC12216819; doi:10.1038/s41598-025-08491-4)
Supplement: Supplementary file 3 — Supplementary Material 3 [file 41598_2025_8491_MOESM3_ESM.docx]

**FigSupp1 Increased NK cell content in tumors of mice treated with blood group A antigens**

The experiment was performed as described in Fig1A. Mice were killed on the 13th day, and tumor tissues and peripheral blood were collected. (A) Flow cytometry was used to detect the proportion of immune cells in mouse tumor tissues. (B) Flow cytometry was used to detect the proportion of immune cells in mouse peripheral blood. (C) Immunohistochemistry was used to detect NK cells in tumor tissues. Immune cell staining scheme in flow cytometry: CD45-PE (30-F11), CD4-FITC (GK1.5), CD8PE-Cyanine7 (53-6.7), NK1.1-APC (PK136). Immunohistochemistry was performed using Anti-NK-1R antibody. The above experiments involved the sampling of quantitative data with mean±SEM, and Student's t-test was used for statistical analysis. At least three replicates were required for each group. *P* < 0.05 was considered statistically significant.
